# The Plague

**Published:** 1898-02-05

**Authors:** 


### THE PLAGUE.

The plague continues to prevail in Bombay, where the deaths, according to latest advices, still number more than 100 per day, which is not far from the level reached at the height of the outbreak last year. Notwithstanding all that has been done, it seems impossible to make sure that all the cases which occur are reported. The condition of affairs in Bombay may best be realised if we bear in mind that the mortality from all causes has risen to 108·7 per 1,000.

Again we have to record rioting in connection with the efforts made by the Government to control the progress of the disease. At Sinnav the hospital assistant was killed, the plague huts and the segregation camp were burnt, and the medical stores and appliances destroyed. To fight the plague is bad enough, but when we have also to contend with prejudice and superstition, and a fanatical hatred of all sanitary measures, it seems almost hopeless to attempt to stand between such a disease and such a people.

